# Ramadan Coincides With the COVID-19 Pandemic: What Should Be Done?

**DOI:** 10.1017/dmp.2020.117

**Published:** 2020-04-22

**Authors:** Seyed Reza Hosseini Zijoud, Alireza Jalali Farahani

**Affiliations:** Atherosclerosis Research Center, Baqiyatallah University of Medical Sciences, Tehran, Iran

The first day of Ramadan in 1441 A.H. will coincide with April 25, 2020. At the time of this writing, in less than a week, Ramadan begins. Almost all over the world, the observation will be overshadowed by the spread of severe acute respiratory syndrome coronavirus 2 (SARS-CoV-2).

On March 11, 2020, the World Health Organization (WHO) announced the outbreak of coronavirus disease 2019 (COVID-19), due to SARS-CoV-2, which began in December 2019, in Wuhan, China, as a pandemic.^1^ As of April 20, 2020, a total of 210 countries and territories around the world and 2 international conveyances were affected, and approximately 2.5 million cases of COVID-19 and approximately 170 thousand deaths (fatality rate, 6.5%) were recorded worldwide.^2^

The outbreak of COVID-19 has, so far, halted many religious rites and ceremonies, and Muslims have been no exception; congregational prayers have been suspended, shrines have been closed, and even the Hajj has been canceled. Now, the month of Ramadan is coming, which is important for Muslims.

Many countries have implemented and extended the state of Health Emergency and Concern to prevent the spread of SARS-CoV-2. During this Ramadan, unlike in previous years, mosques will be closed for the first time in most countries. Traditionally, during the month of Ramadan, mosques are places for Muslims to gather and hold prayers and mass Iftar. In an effort to maintain social distance, all religious gatherings during this month of Ramadan are forbidden, because human gatherings and human-to-human transmissions are the main cause of the spread of the virus.^3^ Interestingly, fasting during Ramadan may reduce human interactions due to a relative decrease in a person’s energy level, which can be helpful in implementing World Health Organization (WHO) protocols in preventing outbreaks of COVID-19.^4^

The month of Ramadan is associated with special rituals for Muslims during the day, including a ban on eating, drinking, smoking, and sexual intercourse for the majority of adults. The pattern of eating changes during Ramadan, and the amount and type of food eaten during the night may also vary.^5^

Some health benefits and potential health concerns of fasting have been addressed in previous studies.^5^ Now, fasting during the relatively hot days of Ramadan poses a challenge to dealing with COVID-19. Long hours of fasting and abstinence from water may lead to dehydration, which is an important health concern because one of the health tips to prevent COVID-19 is to drink water frequently.

It is also recommended to strengthen the body’s immune system, including through healthy and adequate nutrition and drinking fluids, to prevent COVID-19 and lessen the severity of its symptoms. SARS-CoV-2 can cause serious clinical consequences by disrupting the immune system.^3^ However, previous studies have not shown a negative effect of fasting during Ramadan on the immune system; on the contrary, fasting with psychological and spiritual relaxation reduces stress and anxiety.^5^

According to Islamic law, patients or people whose health is endangered by fasting are exempt from fasting on Ramadan. During the COVID-19 pandemic, according to the health recommendations of physicians, as well as the Fatwas, patients with COVID-19 and healthy people who are susceptible to COVID-19 by fasting are exempt from fasting this Ramadan. Also, according to Islamic law, fasting is not obligatory for people with underlying disease, as well as the elderly and people who are physically weak. Various studies have shown that these groups are more vulnerable to COVID-19.^3^ Therefore, fasting is not recommended for these people during this period of COVID-19 pandemic.

In the case of people who have recovered from COVID-19, the health and medical advice is that they should not fast for 6 weeks after recovery, because during COVID-19, the body’s immune mechanisms and electrolyte balances are disrupted and it takes time to return to homeostasis.^3^ So, after 6 weeks, these people can fast if they recover completely.

However, healthy people are advised to fast at home. They can fast during the day, staying at home, with less activity, following health tips, and eating fortified foods and adequate beverages at night. Therefore, all healthy people under the age of 65 can fast by adhering to the following directions: (1) Follow all health advice to prevent COVID-19, such as keeping social distance, clean hands frequently with soap and water, or an alcohol-based hand rub, using masks and gloves, and avoiding contact with patients with COVID-19.^4^ (2) Drink 8 to 10 glasses of water between Iftar and Sahar (at night). (3) Use a nebulizer or boil water in open containers to keep the home environment relatively moist. (4) Avoid hot and dry environments. (5) Reduce consumption of tea, coffee, and sweets and increase consumption of vegetables and fruits between Iftar and Sahar (at night).
